# Continuous Infusion of Dexmedetomidine for Maintenance of Sedation in an Aggressive Adolescent with Autism Spectrum Disorder in the Emergency Department

**DOI:** 10.3390/children11070833

**Published:** 2024-07-09

**Authors:** Giorgio Cozzi, Alessandro Zago, Federico Poropat, Ingrid Rabach, Egidio Barbi, Alessandro Amaddeo

**Affiliations:** 1 Institute for Maternal and Child Health–IRCCS “Burlo Garofolo”, 34137 Trieste, Italy; giorgio.cozzi@burlo.trieste.it (G.C.); federico.poropat@burlo.trieste.it (F.P.); ingrid.rabach@burlo.trieste.it (I.R.); egidio.barbi@burlo.trieste.it (E.B.); alessandro.amaddeo@burlo.trieste.it (A.A.); 2 Department of Medicine, Surgery and Health Sciences, University of Trieste, 34127 Trieste, Italy

**Keywords:** dexmedetomidine, aggressivity, sedation, continuous sedation, pediatric emergency department

## Abstract

Background: The treatment of aggressive behavior and agitation in pediatric patients with autism spectrum disorder (ASD) in the emergency department is topical and challenging. Case Presentation: We described an adolescent with autism spectrum disorder treated ten times in the pediatric emergency department for severe episodes of aggressiveness and agitation. After resolving the acute phase of these behavioural crises, sedation was maintained with a continuous infusion of dexmedetomidine to prevent the resurgence of agitation and to organize discharge properly, considering the family’s needs. The continuous infusion of dexmedetomidine allowed the patient to remain asleep most of the time during his stay at the emergency department. No adverse events were recorded. Conclusions: The continuous infusion of dexmedetomidine could represent a safe and valuable tool to facilitate the permanence of the patient in the PED.

## 1. Introduction

Autism spectrum disorder (ASD) is a pervasive developmental alteration characterized by difficulty in comprehension and communication, with an estimated prevalence of 1% in childhood [1]. Subjects with ASD may present behavioural crises in the form of self- and hetero-aggression and agitation that could be a real challenge for families and healthcare staff in and out of the hospital.

The emergency department (ED) is, indeed, disordered, novel, and rich in auditory and visual stimuli that could trigger or maintain behavioural crises in subjects with ASD. Nevertheless, referring to EDs may represent a unique solution for caregivers to manage aggressive conduct [2]. Children and adolescents with ASD are four times more likely to access pediatric EDs than neurotypical peers [3]. Furthermore, they are prone to receive pharmacological sedation to stop behavioral crises because nonpharmacological de-escalation techniques are frequently unsuccessful [4].

When all the nonpharmacological approaches fail, benzodiazepines, antipsychotic drugs, and ketamine are the sedatives most frequently used to control behavioral alterations in children with ASD [4,5]. Less evidence is available concerning the best procedure after resolving the acute phase of aggressiveness and agitation when immediate discharge from the ED or hospital admission is not feasible. Sometimes, these patients remain boarded in the ED for many hours or even days due to logistic factors, with a significant risk of resurging new episodes of aggressiveness and agitation. Dexmedetomidine is a highly selective alpha-2 agonist whose action mimics natural sleep. Dexmedetomidine shows a slight risk of respiratory adverse events compared to other sedatives [6]. Substantial evidence is now available about dexmedetomidine’s safety and efficacy when used for sedation outside the operating room [7,8], with several reports focusing specifically on patients with autism spectrum disorder [9,10,11,12].

This report describes the benefit of a continuous infusion of dexmedetomidine to maintain sedation in an adolescent with ASD after acute episodes of aggressiveness and agitation in the ED.

## 2. Case Presentation

F. is a 15-year-old, 60 kg adolescent with ASD and epilepsy who was referred ten times, from June 2021 to May 2023, to the Pediatric ED (PED) of the tertiary level university teaching children’s hospital Institute for Maternal and Child Health IRCCS Burlo Garofolo of Trieste, Italy, for episodes of unmanageable aggressiveness and agitation, mainly directed against his parents.

These episodes repeated two to five times yearly over a three-year period despite the considerable effort made by the hospital and territorial healthcare services to prevent them.

The episodes were mainly triggered by the change of environment when F. was moved from home to daycare or vice versa. Usually, he refused to walk, sitting on the ground and then becoming aggressive, hitting his parents and destroying objects. Other times, agitation and aggressiveness started at home. Parents were trained to perform nonpharmacological de-escalation strategies and to implement oral antipsychotic therapy when needed, according to the prescription of the territorial child psychiatrists.

Most of the time, parents were able to contain the episodes, but when all the strategies failed and agitation and aggressiveness were too severe, they alerted the emergency medical service (EMS). EMS operators tried to manage the episodes as well, but sometimes they needed to recur to parenteral pharmacological sedation and to refer the adolescent to the PED. In these cases, F. arrived deeply sedated or still combative. Remarkably, he had previous admissions to the intensive care unit (ICU), during which he was kept sedated with a continuous infusion of propofol. Frequently, parents presented bruises and scratches. They reported that the severity and dangerousness of the episodes worsened with F.’s age despite antipsychotic therapy. They were exhausted and not able to rehost their son promptly.

When at the emergence of sedation F. was again aggressive and agitated, not responding to nonpharmacological techniques, and refusing oral medications, in agreement with his parents, continuous sedation with dexmedetomidine was implemented to avoid the further resurging of agitation and to facilitate his permanence in the PED. The sedation aim was to maintain the patient inside a Ramsay sedation score between 3 and 4 most of the time.

We reviewed the medical records of the episodes in which the continuous infusion of dexmedetomidine was used.

Table 1 shows the pharmacological treatment that the patient received for his aggressiveness and agitation. The drugs were administered mainly out of the hospital. Sometimes, acute therapy was implemented upon arrival at the PED. Moreover, it reveals the doses of dexmedetomidine employed during the continuous infusion and the time between the sedative’s suspension and the patient’s discharge.

The attending pediatrician decided the dose of dexmedetomidine and modulated it according to the patient’s response. Usually, the infusion ranged from 0.5 to 1 mcg/kg/h. The patients remained asleep most of the time, but arousable. He was monitored continuously with pulse oximetry and cardio monitor, and an operator was always with him to let the parents rest at home. Monitoring never indicated any significant change in cardiac or respiratory parameters needing medical intervention. Hydration was provided parenterally, and eating was not allowed. Most of the usual pharmacological therapy was provided parenterally as well. Among the episodes, sedation lasted between 12 and 28 h. No adverse events were recorded during sedation, and no resurgence of agitation or an escalation of pharmacological therapy or any advanced support was needed. On one occasion, the attending pediatrician was concerned about a possible urine retention and decided upon urethral catheterization.

Despite F.’s crises being clearly linked to specific situational triggers, a careful clinical evaluation and a series of laboratory tests were carried out to look for organic causes of the increased agitation.

The patient was always discharged at home from the PED. According to the parents, the ED pediatrician decided the end of the sedation to favor their logistic needs. The tapering of sedative was brief, as it was the time from the suspension of sedation and discharge (at least one hour). At discharge, F. was judged alert, eupneic, and able to stand and walk autonomously.

Parents reported no adverse events at home after discharge. Moreover, no readmissions within 24 h from discharge occurred.

From episode to episode, repeated meetings were performed with parents, PED and ICU staff, EMS operators, and hospital and territorial child psychiatrists to explore different solutions, improve logistical management, pharmacological baseline therapy, and implement nonpharmacological and pharmacological strategies to manage acute agitation and aggressiveness, trying to avoid recurrence of continuous pharmacological sedation as much as possible.

## 3. Discussion

The safety and efficacy of dexmedetomidine sedation outside the operating room and the ICU is already well established. Instead, to our knowledge, this is the first report describing the repeated use of a continuous infusion of dexmedetomidine for the maintenance of sedation after episodes of severe agitation and aggressiveness in patients with ASD in the ED setting. ED management of ASD patients represents a very challenging and emergent problem considering the actual prevalence of the disease.

Evidence regarding the management of acute behavioural crises in subjects with ASD suggests the use of nonpharmacological de-escalation techniques as a first-line treatment. However, when these techniques are insufficient, pharmacological therapy is indicated. The drugs frequently used in the PED setting are benzodiazepines, antipsychotics, and ketamine [4,5,13,14].

Behavioural interventions and oral medical therapies work most of the time without the need for parenteral sedation and should always be tried. In severe cases, a prompt psychiatric admission should be attempted as well.

On the other hand, these strategies may fail or may not be feasible.

There is increasing evidence and attention to the use of dexmedetomidine in the PED setting [15,16]. However, its utilization is mainly described when administered through the intranasal route, to perform brief diagnostic tests or to decrease agitation during respiratory distress needing ventilatory support [12,16,17,18].

Despite the continuous infusion of dexmedetomidine being a commonly employed treatment in the ICU setting to maintain sedation in children [19] and to perform brief procedures outside the operating room [7,8], no previous reports describe its application for continuous sedation in the PED setting.

Evidence from the ICU setting proves that the prolonged continuous infusion of dexmedetomidine is safe, with types and rates of adverse events similar to that reported for short infusion. In particular, bradycardia and hypotension are expected but rarely need medical intervention [20,21].

We have repeatedly asked ourselves and discussed whether keeping an adolescent with a neuro-atypical condition under sedation for several hours in the PED was reasonable or ethically acceptable, safe for him, and whether it was an inappropriate use of the ED space and resources to try to help F. and his family.

In these limited cases, it is difficult to draw a clear line of what is ethical or unethical. Continuous pharmacological sedation may be perceived as an unethical form of pharmacological restraint, but how can we be sure that other, more standardized, and commonly performed approaches are, in the end, more ethical than this one? Remaining blocked in an unfamiliar and frightening environment, passing through a series of antipsychotics and sedatives, frequently injected several times in a few hours, could be perceived as unethical as well.

Continuous pharmacological sedation should be considered only in settings with the appropriate monitoring, staff availability, and appropriate training to guarantee the patient’s safety; therefore, it is usually limited to ICU and intermediate care unit settings. Conversely, an equipped ED may offer advantages compared to an ICU such as more straightforward and open access for parents and a more convenient setting for awakening with less monitoring, noise, and lights.

Indeed, even if required in highly selective cases, continuous pharmacological sedation could still be perceived as a too-invasive and disrespectful approach to prevent the resurgence of agitation and aggressiveness.

In this sense, dexmedetomidine is a versatile drug with a higher safety profile compared to propofol and ketamine. It induces a state resembling natural sleep, making the patient easily arousable and supporting spontaneous ventilation, airway tone, and pulmonary function. Furthermore, it decreases the incidence of delirium after anesthesia and has a short recovery time [22]. Therefore, these characteristics make this drug an attractive choice for organizing the permanence and the discharge of aggressive patients who need prolonged sedation and fast recovery with a low risk of adverse events before discharge.

As a single case with all the possible confounding variables, we cannot provide new solid indications or suggest this approach as a new strategy whenever less invasive solutions are feasible.

We have always decided to recur to the continuous sedation of dexmedetomidine as an “extrema ratio”. However, this report highlights that this choice may be an option that can be at least considered until other solutions are found. While in a general perspective physicians may feel uncomfortable housing spontaneous-breathing continuously sedated patients, this paper highlights how this may be considered feasible using dexmedetomidine with a standard PED monitoring.

## 4. Conclusions

In conclusion, this report suggests a possible role for continuous infusion of dexmedetomidine in maintaining sedation in an aggressive adolescent with ASD not responding to other strategies. In selected cases, this approach may facilitate the patient’s permanence in the PED and help the operators and the family to organize the discharge as best they can.

## Figures and Tables

**Table 1:** Pharmacological therapy is employed in the patient described in the report.

Visits	Pharmacological Treatment for Aggressiveness and Agitation Performed out of Hospital and in the ED	Maintenance of Sedation with Continuous Infusion of Dexmedetomidine			
	Drugs and Dose	C.I. Dose (mcg/kg/h)			Time between Stop of Sedation and Discharge
14/06/2021	Clotiapine 12 gtt PO	Start	0.5	1 h	2 h
	Risperidone 2 mg PO	Max	1	13 h	
1	Propofol IV 170 mg	Tapering	0.5	1 h	
18/08/2021	Clotiapine 12 gtt PO	Start	0.5	3 h	2 h
	Risperidone 2 mg PO	Max	0.9	14 h	
	Lorazepam 2 mg PO	Tapering	0.5	1 h	
	Promazine 100 mg IM				
2	Ketamine 100 mg IM				
13/05/2022		Start	0.3	7 h	1 h
	Clotiapine 10 gtt PO	Max	0.7	20 h	
	Midazolam 5 mg IV	Tapering	0.5	1 h	
	Ketamine 50 mg IV				
3					
14/05/2022	Midazolam 10 mg IV Ketamine 50 mg IV	Start	0.5	3 h	1 h
		Max	0.5	16 h	
		Tapering	0.5	1 h	

4					
24/05/2022	Midazolam 5 mg IN Midazolam 2 mg IV Lorazepam 5 mg IV Ketamine 50 mg IV	Start	0.5	2 h	1 h
		Max	0.7	12 h	
		Tapering	0.5	1 h	

5					
24/09/2022	Midazolam 2.5 mg IV	Start	0.5	2 h	1 h
	Midazolam 5 mg IV	Max	1	16 h	
	Midazolam 0.3 mg/kg/h C.I.	Tapering	0.5	1 h	
6					
13/12/2022	Ketamine 100 mg IV	Start	0.3	1 h	1 h
	Ketamine 60 mg IV	Max	1	23 h	
	Propofol 60 mg IV	Tapering	0.5	1 h	
7	Dexmedetomidine 30 mcg IV				
16/01/2023	Midazolam 5 mg IV	Start	0.5	1 h	1 h
	Midazolam 5 mg IV	Max	1	11 h	
	Propofol 20 mg IV	Tapering	0.5	1 h	
8	Midazolam 0.1 mg/kg/h C.I.				
	Clotiapine 20 gtt PO	Start	0.5	1 h	1 h
	Midazolam 2 mg IV	Max	1	10 h	
4/02/2023	Midazolam 3 mg IV	Tapering	0.5	1 h	
	Midazolam 4 mg IV				
9	Ketamine 20 mg IV				
	Ketamine 20 mg IV				
	Midazolam 0.1 mg/kg/h C.I.				
	Midazolam 15 mg PO	Start	0.3	1 h	1 h
23/05/2023	Clorpromazine 50 mg PO	Max	0.7	11 h	
	Midazolam 3 mg IV	Tapering	0.5	1 h	
10	Dexmedetomidine				
	50 mcg IV in 10′				

## Data Availability

Data is contained within the article.
